# Robust Time-Difference-of-Arrival (TDOA) Localization Using Weighted Least Squares with Cone Tangent Plane Constraint

**DOI:** 10.3390/s18030778

**Published:** 2018-03-04

**Authors:** Bonan Jin, Xiaosu Xu, Tao Zhang

**Affiliations:** Key Laboratory of Micro-Inertial Instrument and Advanced Navigation Technology, Ministry of Education, School of Instrument Science and Engineering, Southeast University, Nanjing 210096, China; jinbonan@seu.edu.cn (B.J.); 101011356@seu.edu.cn (T.Z.)

**Keywords:** cone, positioning, robust, Taylor, TDOA, WLS

## Abstract

Finding the position of a radiative source based on time-difference-of-arrival (TDOA) measurements from spatially separated receivers has been widely applied in sonar, radar, mobile communications and sensor networks. For the nonlinear model in the process of positioning, Taylor series and other novel methods are proposed. The idea of cone constraint provides a new way of solving this problem. However, these approaches do not always perform well and are away from the Cramer-Rao-Lower-Bound (CRLB) in the situations when the source is set at the array edge, the noise in measurement is loud, or the initial position is biased. This paper presents a weighted-least-squares (WLS) algorithm with the cone tangent plane constraint for hyperbolic positioning. The method adds the range between the source and the reference sensor as a dimension. So, the space-range frame is established. Different from other cone theories, this paper sets the reference sensor as the apex and finds the optimal source estimation on the cone. WLS is used for the optimal result from the measurement plane equations, a vertical constraint and a cone constraint. The cone constraint equation is linearized by a tangent plane. This method iterates through loops and updates the tangent plane, which approximates the truth-value on the cone. The proposed algorithm was simulated and verified under various conditions of different source positions and noises. Besides, some state-of-the-art algorithms were compared in these simulations. The results show that this algorithm is accurate and robust under poor external environment.

## 1. Introduction

Location technology based on time of arrival is an important branch of passive location and crucial in radar searching, underwater positioning, indoor positioning and other similar applications. As an object-detection system, radar determines the range, angle, or velocity of objects by radio waves. Radio waves (pulsed or continuous) from the transmitter reflect off the object and return to the receiver, give information about the object’s location and speed [1]. It thus can be used to detect aircraft, ships, spacecraft, guided missiles, motor vehicles, weather formations and terrain. When an autonomous-underwater-vehicle (AUV) is executing a task in deep sea without GPS or other radio signals, Long-base-line (LBL) acoustic positioning system is effective in AUV navigation. LBL gathers distance information between the underwater objective and the seabed array element to solve the target position. It can provide accurate positioning of an underwater vehicle within a local area [2]. Indoor positioning also needs the support of this technology. Reference [3] presents an ultrasonic-based indoor positioning (ICKON) system for indoor environments. It uses only ultrasonic signals and calculates the position of the mobile platform at centimeter-level accuracy. Reference [4] considers the use of seismic sensors for footstep localization in indoor environments based on TDOA. The position estimation error is only some tens of centimeters. In addition, the strategies of smartphone localization and speaker localization are analogous to indoor positioning [5,6]. The technology is also widely applied in radio navigation, such as the famous LORAN system and Gee system. When the earthquake occurs, earthquake waves inside of radio signal and provides the TDOA measurements in seismic source finding.

TDOA is one of the means of passive location. Two basic sensors (BSs) serves as the focus. The difference between the distances of the two BSs to a source serves as the focal length, which makes up a hyperbola. The intersection of two hyperbolas provides the source position. Compared to time-of-arrival (TOA), TDOA localization does not need synchronization between the source and receivers and are easy to implement [7,8]. It eliminates system errors to a certain extent, improves location precision and performs with high accuracy in non-line-of-sight (NLOS) environments [9]. Solving nonlinear equations is a Gordian knot in TDOA localization due to a sum of squares of coordinates and the range. Numerous methods have been introduced, validated and published in the literature. The most used TDOA-based source-localization algorithms when LOS between receivers and sources exist are summarized in [10]. 

Common closed-form solution includes Fang, SI, SX and Chan [11,12,13]. Fang uses an elimination method to multivariate nonlinear equation into a quadratic equation and then solves unknowns one by one. However, there may be no root or multiple roots in the quadratic equation. The problems of computation and root are more serious in 3-D. In addition, the number of equations must equal to the number of unknowns. Therefore, precision cannot be improving by comprehensively analyzing the redundant information from more BSs. SI assumes the source’s coordinates *x*, *y* and the range *r*_0_ between the source and the reference sensor are independent of each other and then eliminates *r*_0_. It does not consider the geometrical relationship of the source coordinates and range. So, SI performs well in distant but dissatisfactory in close. SX assumes known *r*_0_ and solves *x*, *y* in terms of *r*_0_. And then it uses *r*_0_ to find *x*, *y*. Since *r*_0_ is assumed constant in the first step, the degree of freedom to minimize the second norm of *ϕ* is reduced. The solution obtained is, therefore, non-optimum [14]. Chan is widely used with two features. One assumes *x*, *y* and *r*_0_ are independent and obtains the optimal estimate by WLS. The other is a correction with increment square. However, there are some flaws in the process. The first feature will lead to a contradiction among *x*, *y* and *r*_0_ especially in distant, which affects the other relative values later. The second feature gets multi results after being square rooted. The correction is uncorrelated with geometric equations, so it is hard to find a balance between the result of correction and geometric solution. In addition, Chan proposes two approaches to solve the problem in the different range. Nevertheless, the critical point is not specific. 

The iterative method tends to be more accurate than the closed-form solution. It requires an initial value to begin the process. The result is challenged by the initial value quality and the final convergence effect. The Taylor estimator is such an effective method [15] but it suffers from local convergence [16]. Reference [17] offers a new and unifying perspective that includes the adoption of a multidimensional reference frame obtained by adding the range difference coordinate to the spatial coordinates of the source. Reference [18] proposed a localization approach through the fitting of propagation cones (FoPC). It also constructed a space-range reference frame with range as the third axis. All of the sensors in the array correspond to the points on the cone in this frame. The apex of the cone is the source position. It transforms the hyperbolic localization into finding the optimal cone based on the sensors for the source position. The optimal cone is from iterative updating using maximum likelihood estimation. Reference [19] has done more research based on this idea.

Inspired by these perspectives, this study adds the range as a new coordinate to construct a multidimensional reference frame, which consequently facilitates linearization of the surface problem from the perspective of spatial geometry. Unlike [18], the reference sensor is set to be the cone’s apex in this method. So, the apex and the cone are fixed, the source to be known is on the cone surface. We use WLS to find the optimal source position. WLS is an optimal estimation of linear problems. In this paper, besides the measurement planes in the basic mathematical model, WLS includes a vertical plane constraint and a cone tangent plane constraint. The cone tangent plane constraints the range-coordinates relationship instead of the cone in local approximation. The vertical constraint and the cone tangent constraint are derived from the initial value and updated again after each iteration. The result is close to the optimal solution on the cone by multiple WLS.

In the following sections, we will introduce the algorithm in detail and compared with Taylor algorithm and FoPC proposed in [18] under different conditions. In some locations, Taylor and FoPC may be divergent. The simulations section shows the characteristics comparisons of these methods. It illustrates that the approach in this paper performs better than the state-of-the-art algorithms do.

## 2. Algorithm and Process

### 2.1. Problem Description

The source sends the signal *X*(*t*), *N* + 1 BSs receive *N* + 1 signals *Y_i_*(*t*), where *i* = 0, 1, 2, 3, …, *N* is the number of BS, No. 0 BS is the reference. Usually, cross-correlating BS signal *Y_i_*(*t*) (*i* = 1, 2, 3 … *N*) with BS_0_ signal *Y*_0_(*t*) can get *N* cross-correlation functions *R_i_*_0_(*τ*) (*i* = 1, 2, 3, …, *N*). After detecting peaks on *R_i_*_0_(*τ*) and eliminating the interference from ambiguous peaks with prior information [20], the signal time difference of arrival measurements *τ_i_*_0_ (*i* = 1, 2, 3 … *N*) between BSi to BS_0_ is found.

Set the source position as ***p*** = [*x, y*]*^T^* and No. *i* BS position is set as ***s****_i_* = [*x_i_, y_i_*]*^T^* (*i* = 1, 2, 3, …, *N*). The distance relationship (range *r_i_* and position ***p***) between the source and BSi is

(1) $$r_{i}^{2}={\left(x-x_{i}\right)}^{2}+{\left(y-y_{i}\right)}^{2},\;i=0,1,2,\ldots ,N.$$

Especially, when *i* = 0, *r*_0_ is the range between the source and the reference sensor BS_0_

(2) $$r_{0}^{2}={\left(x-x_{0}\right)}^{2}+{\left(y-y_{0}\right)}^{2}.$$

In the LOS environment, we assume signal velocity constant *c*. Denote {*}*_ij_* = {*}*_i_* − {*}*_j_*, the range difference *r_i_*_0_ is obtained,

(3) $$r_{i0}=c\tau_{i0}=r_{i}-r_{0},\;i=1,2,3,\ldots ,N.$$

**Figure 1:**
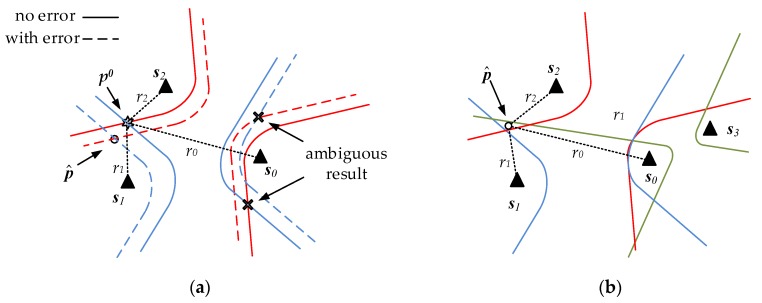
2D hyperbolic localization (each color represents a couple of hyperbolas); (**a**) difference between ideal condition and reality; (**b**) multiple hyperbolas for the optimal position.

The solution of each equation in Equation (3) is on the hyperbola of which ***s****_i_* and ***s***_0_ are the focal points and *r_i_*_0_ is the focal length. The intersection of *N* hyperbola equations exists for ***p***. The solution of every two equations is the intersection of two hyperbolas (Figure 1). We need two equations for two unknowns in the condition without errors. Fang gave an exact solution when the number of TDOA measurements is equal to the number of unknowns. However, the hyperbolas may not always cross at the true position because of errors in time difference measurements. The other intersections will bring ambiguous results (Figure 1a). Thus, more than four BSs are needed to construct over three equations. Though the multiple intersections do not coincide in the same place under error conditions, we can get the optimal position with appropriate estimation (Figure 1b). Consequently, if in 3D hyperboloid localization, the number of measurements is more than four according to five BSs or more.

From the equations above, *r_i_*^2^ = (*r_i_*_0_ + *r*_0_)^2^, *i* = 1, 2, 3, …, *N*. Equation (1) is rewritten as

(4) $${\left\|s_{i}\right\|}^{2}+{\left\|p\right\|}^{2}-2s_{i}{}^{T}p=r_{i0}{}^{2}+2r_{i0}r_{0}+r_{0}{}^{2},\;i=0,1,2,\ldots ,N,$$

when *i* = 0, *r_i_*_0_ = 0. Thus

(5) $${\left\|s_{0}\right\|}^{2}+{\left\|p\right\|}^{2}-2s_{0}^{T}p=r_{0}^{2}$$

Subtract Equation (4) by (5), the hyperboloid equation is obtained

(6) $${\left\|s_{i}\right\|}^{2}-{\left\|s_{0}\right\|}^{2}-2s_{i0}{}^{T}p=r_{i0}{}^{2}+2r_{i0}r_{0},\;i=1,2,3,\ldots ,N.$$

Considering the noise in real situations, *τ_i_*_0_ *=* *τ_i_*_0_^0^ *+ ϕ_i_*, *τ_i_*_0_^0^ is the TDOA truth value, *φ_i_* is the measurement error. We adjust Equation (6) and write it in a matrix form as follows:

(7) $$G_{a}\left[\begin{array}{c} x\\ y\\ r_{0} \end{array}\right]=h_{a}+\Phi_{a}$$

where

(8) $$G_{a}=\left[\begin{array}{cc} s_{10}{}^{T} & r_{10}\\ s_{20}{}^{T} & r_{20}\\ \vdots & \vdots \\ s_{N0}{}^{T} & r_{N0} \end{array}\right],\;h_{a}=\frac{1}{2}\left[\begin{array}{c} K_{10}-r_{10}{}^{2}\\ K_{20}-r_{20}{}^{2}\\ \vdots \\ K_{N0}-r_{N0}{}^{2} \end{array}\right],\;\Phi_{a}=-c\left[\begin{array}{c} r_{1}\varphi_{1}\\ r_{2}\varphi_{2}\\ \vdots \\ r_{N}\varphi_{N} \end{array}\right]=-cB_{a}\sigma ,\;B_{a}=\left[\begin{array}{cccc} r_{1} & 0 & \cdots & 0\\ 0 & r_{2} & \cdots & 0\\ \vdots & \vdots & \ddots & \vdots \\ 0 & 0 & \cdots & r_{N} \end{array}\right]$$

Φ*_a_* is the error ignored higher-order term and ***σ*** is the TDOA measurement error; and *K_i_*_0_ *= K_i_ − K*_0_, *K_i_* = ‖***s****_i_*‖^2^ = *x_i_^2^ + y_i_^2^*, *i =* 0, 1, 2, 3, *…*, *N*.

The equations are linear under the assumption that ***p*** and *r*_0_ are independent of each other [13] but the truth is not as such due to Equation (2). The Chan algorithm calculates ***p*** and *r*_0_ with three independent unknowns and corrects them with Equation (2). Thus, an inevitable discrepancy exists between ***p*** and *r*_0_. If this discrepancy is corrected simply without partiality, then the result must be in accord with Equation (2) but diverges away from the optimal solution. 

### 2.2. Space-Range Frame

**Figure 2:**
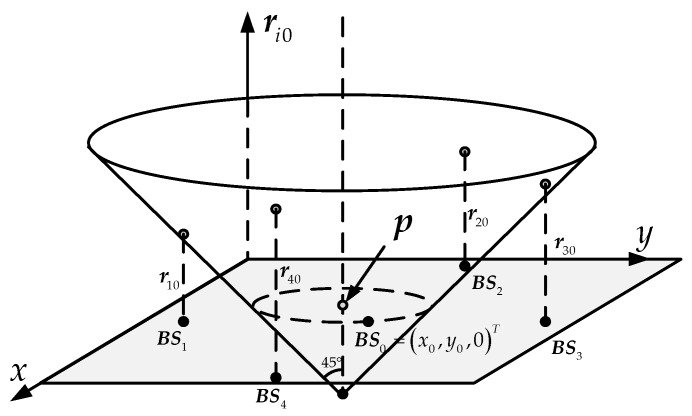
Localization diagram of FoPC algorithm using the space-range frame, as in [18].

Reference [18] assumes that the *z*-axis represents the source-to-sensor range *r_i_* and the source position is set as (*x*_s_, *y*_s_, 0) in the space-range frame. However, the range *r_i_* is unobtainable from TDOA measurements because of non-synchronization, the coordinates of the sensors are hard to define in this space. We suggested the reference sensor coordinates as (*x*_0_, *y*_0_, 0) and the *z*-axis as the TDOA vector. In this definition, the set of points that correspond to a given range lie on a circle that expands as the range increases, forming a cone whose apex is the actual location of the sourced, as Figure 2. Besides, from Equation (1) we can see the cone’s angular aperture is 45° and all the corresponding points of the sensors are located on this cone ideally. In fact, due to the errors, the sensors coordinated in the frame scatter near the real cone. Based on the TDOA measurements and the location of the sensors, the FoPC uses maximum-likelihood estimator (MLE) to get the best cone, thus obtaining the apex, which is the source.

**Figure 3:**
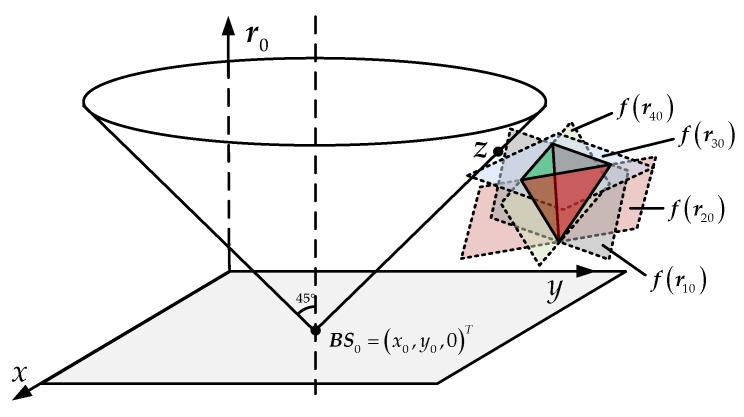
A different localization description with the space-range frame.

Instead of [18], we construct a frame and a cone according to Equation (2), where the *z*-axis represents the source-to-reference-sensor range *r*_0_ and the apex of the cone is set as the reference sensor (*x*_0_, *y*_0_, 0), as Figure 3. The source is thus located on this cone and marked ***z*** = (***p****^T^*, *r*_0_)*^T^*. If the location parameter of reference sensor is accurate, the domain (cone) where the source may be on is unique and independent of system error. Equation (6) is also represented as a cluster of planes in the frame. Ideally, there is only one intersection on these planes, which is the source. However, taking into account the measurement error, the planes intersections are more than one, only enveloping a space with the highest probability of the source position. According to these planes, the enveloped space and the statistical characteristics of the measurement error, there is a certain probability distribution of source locations in the frame. Each point on the cone also has a possibility of being the source and the points where are closer to the envelope are more likely the source. In this idea, we attempt to find the point on the cone surface which may be the source with the maximum probability.

### 2.3. Cone Tangent Plane Constraint

We hope that the cone constraint applies to WLS as the cluster of planes but it is not the case because the cone is nonlinear. Approximating the cone surface a plane locally, we can use WLS on the tangent plane instead of the cone constraint in a region. 

For a given initial position of source (*x*_s_, *y*_s_)*^T^*, it is always on the cone and the tangent plane is determined there. Combined with measurement equations, there will be a cluster of *N* + 1 planes and the new position corrected can be obtained by WLS. If the tangent plane matches the true plane or the gap is small, the estimation is optimal. Otherwise, the result is suboptimal but the gap is shrunken. Repeat the operation several times this way, the estimation will be close to the true location eventually. 

**Figure 4:**
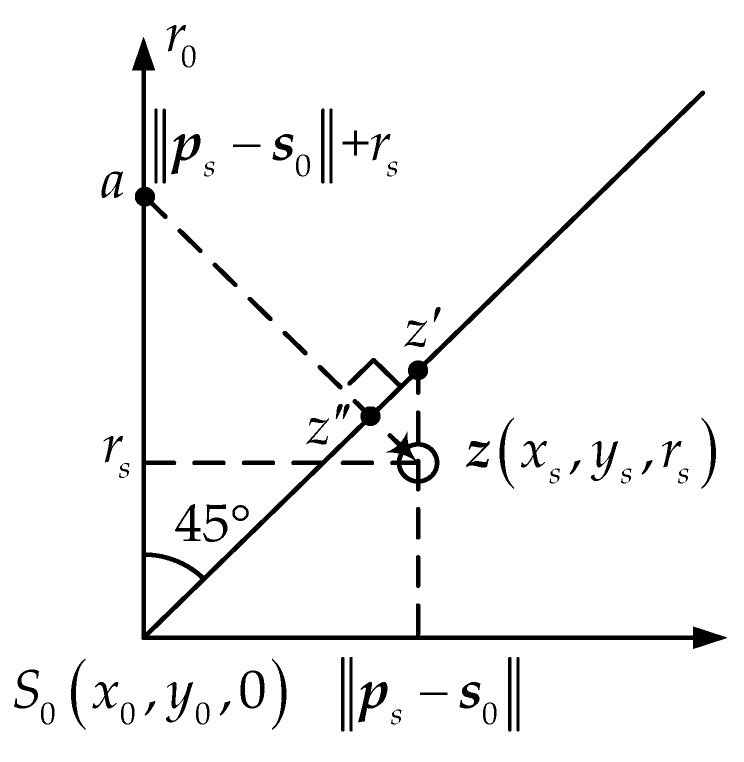
Normal vector longitudinal section.

For the initial position (*x*_s_, *y*_s_, *r*_s_)*^T^* containing the range information, such as the WLS answer, it may deviate from the cone surface due to errors. See in Figure 4, the tangent plane is obtained from the point *z*’ located at (*x*_s_, *y*_s_)*^T^* or *z*’’ where is closest to the initial point on the cone. Anyway, the result is equivalent.

The tangent plane, whose normal vector passing through ***z*** and the *r*_0_ axis, is identified as the local approximation of the cone [14] and viewed as the nearest place to ***z***. As shown in Figure 4, the normal vector ***az*** is available

(9) $$az=\left(x_{s},y_{s},r_{s}\right)-\left(x_{0},y_{0},\left\|p_{s}-s_{0}\right\|+r_{s}\right)=\left(p_{s}{}^{T}-s_{0}{}^{T},-\left\|p_{s}-s_{0}\right\|\right).$$

The tangent plane is

(10) $$\left[\begin{array}{cc} p_{s}{}^{T}-s_{0}{}^{T} & -\left\|p_{s}-s_{0}\right\| \end{array}\right]\cdot \left[\begin{array}{c} x-x_{0}\\ y-y_{0}\\ r_{0} \end{array}\right]=0.$$

**Figure 5:**
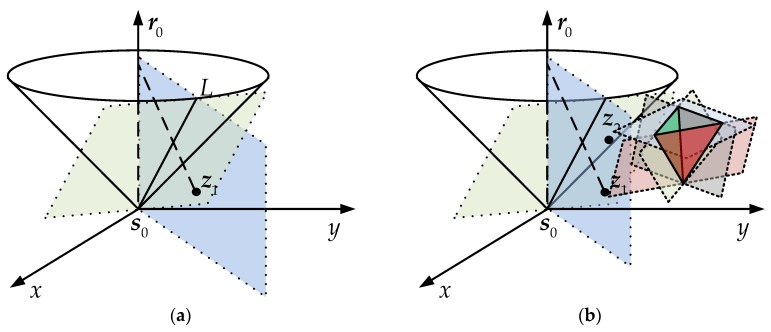
Cone tangent plane constraint localization. (**a**) The cone tangent plane and vertical plane, (**b**) the correction with WLS.

The intersection of the tangent plane and the cone surface, line *L*, is the area with the highest linear approximation. The position away from *L* on the tangent plane is no longer suitable for the cone equivalent. We set up a vertical plane through *r*_0_ axis and ***z***, which intersects the tangent plane at *L*, Figure 5a. It is also taken into account when doing WLS, which constrains the estimation from deviating from *L*. Without this plane, the estimation may fall into a singular point and thus fail to converge. The vertical plane is

(11) $$\left[\begin{array}{ccc} y_{s} & -x_{s} & 0 \end{array}\right]\cdot \left[\begin{array}{c} x\\ y\\ r_{0} \end{array}\right]=0.$$

Figure 5b is the final plane cluster for WLS, ***z***_1_ is the initial value and ***z***_2_ is the result of one correction. Compared with ***z***_1_, ***z***_2_ is closer to the cone and its sum of mean square errors to the cluster of measurement planes is modest.

### 2.4. The Weight of the Constraints

The WLS method requires the exact weight of each linear equation to obtain the best estimate. The weighted matrix of measurement plane clusters is obtained from Equation (8) [13].

(12) $$\psi_{measure}=E\left(\Phi_{a}\Phi_{a}{}^{T}\right)=c^{2}B_{a}\cdot Q\cdot B_{a},\;Q=\sigma \sigma^{T},$$

where *B_a_* is calculated by the initial position.

**Figure 6:**
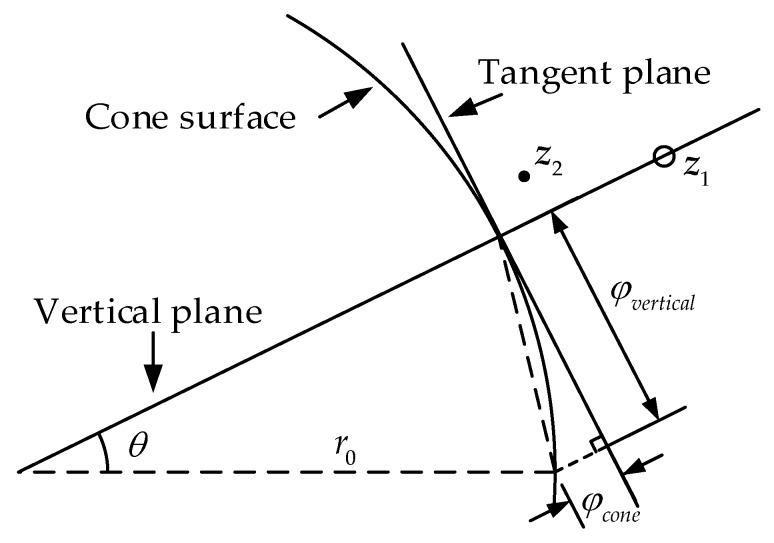
The weight distance diagram of the constraints.

The WLS solution is a point of weighted least distances to planes. The tangent and vertical weighted distances *φ_vertical_* and *φ_cone_* are defined as shown in Figure 6. In the range of ±*θ*, it is considered that the tangent plane and the cone surface are equivalent.

*φ_vertical_* and *φ_cone_* are easily resolved

(13) $$\varphi_{vertical}=r_{0}\sin\theta ,\;\varphi_{cone}=r_{0}(1-\cos\theta )=r_{0}\frac{1-\cos^{2}\theta }{1+\cos\theta }\approx r_{0}\frac{\sin^{2}\theta }{2}$$

when *θ* is small, the approximation equation is established. If *θ* is too small, it affects the convergence rate. If *θ* is too large, the equivalence of the cone and the tangent is poor. sin*θ* = 0.045 is set in this paper.

Assumed the tangent plane and vertical plane independent each other and they are independent with measurement planes. Thus, the final covariance matrix is

(14) $$\psi =\left[\begin{array}{ccc} \psi_{measure} & 0 & 0\\ 0 & \varphi^{2}{}_{cone} & 0\\ 0 & 0 & \varphi^{2}{}_{vertical} \end{array}\right].$$

### 2.5. Iteration and Final Result

All the planes and weights are ready and the final weight is now available.

(15) $$G_{b}\left[\begin{array}{c} x\\ y\\ r_{0} \end{array}\right]=h_{b}+\Phi_{b},$$

where

(16) $$G_{b}=\left[\begin{array}{cc} s_{10}{}^{T} & r_{10}\\ s_{20}{}^{T} & r_{20}\\ \vdots & \vdots \\ s_{N0}{}^{T} & r_{N0}\\ {\left(p_{s}-s_{0}\right)}^{T} & -\left\|p_{s}-s_{0}\right\|\\ \left[\begin{array}{cc} y_{s} & -x_{s} \end{array}\right] & 0 \end{array}\right],\;h_{b}=\frac{1}{2}\left[\begin{array}{c} K_{10}-r_{10}{}^{2}\\ K_{20}-r_{20}{}^{2}\\ \vdots \\ K_{N0}-r_{N0}{}^{2}\\ 2{\left(p_{s}-s_{0}\right)}^{T}s_{0}\\ 0 \end{array}\right],\;\Phi_{b}=\left[\begin{array}{c} \Phi_{a}\\ \varphi_{cone}\\ \varphi_{vertical} \end{array}\right].$$

The optimal estimation is

(17) $${\left(x,y,r_{0}\right)}^{T}={\left(G_{b}{}^{T}\psi^{-1}G_{b}\right)}^{-1}G_{b}{}^{T}\psi^{-1}h_{b}.$$

**Figure 7:**
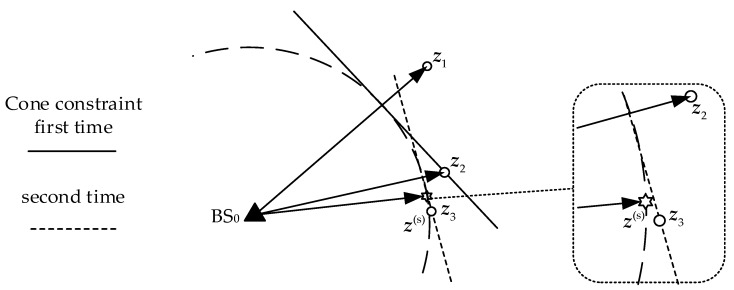
The position change after twice Cone constraint correction.

The optimal point of these planes is obtained by WLS easily. The result is closer to the cone after the correction several times. The position result after the correction is more accurate than that of the first estimation. It is able to construct a more accurate cone constraint. Therefore, the correction can be operated repeatedly using the result in the last step according to the need. As Figure 7, ***z***_1_ is the initial position. ***z***_2_ is the estimation after the correction first time and set as the start for the next correction. ***z***_3_ is the position result after twice correction. The iterative process will continue until the increment from the correction is less than the threshold.

## 3. Performance Analysis

In this section, some simulations were run for the performance of the cone constraint algorithm. Notice that the huge performance advantage of the iterative approaches over the closed-form algorithms such as Chan and SI. Therefore, the proposed algorithm is compared with Taylor and FoPC. In the FoPC, two methods, *Jεa* and *Jεe*, are proposed based on different cost functions [18]. Their differences are defined in Equations (18) to (21).

(18) $$\hat{X}=\arg\min\left(J_{\varepsilon k}\right)=\arg\min\left(\varepsilon_{k}{}^{T}\varepsilon_{k}\right),$$

where

(19) $$\varepsilon_{k}={\left[\begin{array}{cccc} \varepsilon_{k,0} & \varepsilon_{k,1} & \cdots & \varepsilon_{k,N} \end{array}\right]}^{T},$$

and the subscript *k* is replaced by *a* or *e* according to which error definition is adopted.

(20) $$\varepsilon_{e,i}={\left(x_{i}-x\right)}^{2}+{\left(y_{i}-y\right)}^{2}-{\left(r_{i}-r\right)}^{2},$$

(21) $$\varepsilon_{a,i}=\sqrt{{\left(x_{i}-x\right)}^{2}+{\left(y_{i}-y\right)}^{2}}-\left(r_{i}-r\right).$$

We start introducing the metrics adopted for accuracy evaluation and then we describe the simulation setup. The comparison of robustness is analyzed in the second section. Some of the factors, which affect positioning performance, were simulated then. The first is the accuracy analysis when the source at different locations. Another is TDOA errors, we tested the impact of different degrees of noise on TDOA localization.

### 3.1. Setup and Evaluation Metrics

**Figure 8:**
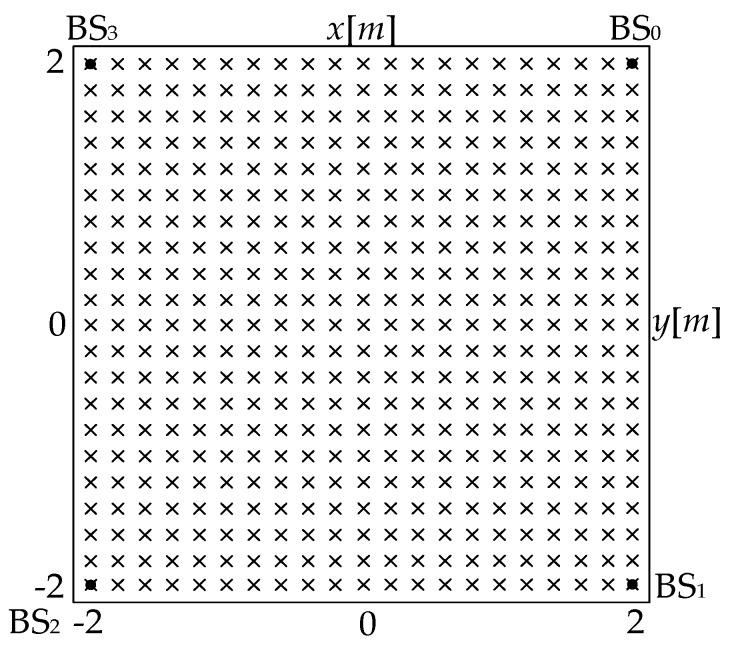
Setup of the source and BSs.

As Figure 8, the setup used for simulations and experiments is as same as that of the single array simulation in [18]. The filled circles denote BSs, while crosses mark possible positions of the source. In particular, the area covered by BSs in the simulations is a square of 4 m × 4 m. We assume that BS locations are calibrated, i.e. their positions are not affected by errors.

The time difference was simulated as truth added with Gaussian white noise. Considering that a single test is contingent, the Monte Carlo method was performed with various inputs. The statistical results of 200 tests in each input condition (location or noise) were illustrated the performance. The accuracy of the localization algorithm is evaluated using the following metrics, they are also used in [18] and we improve some definitions.

Average bias of the localized source is

(22) $$b=\sqrt{b_{x}{}^{2}+b_{y}{}^{2}},\;b_{x}=\frac{1}{n}\sum_{i=1}^{n}\left(x-{\hat{x}}_{i}\right),\;b_{y}=\frac{1}{n}\sum_{i=1}^{n}\left(y-{\hat{y}}_{i}\right),$$

where *n* is the number of noisy measurements tested for each source location, *x* and *y* are the coordinates of the source, ${\hat{x}}_{i}$ and ${\hat{y}}_{i}$ are their estimations based on the *i*th realization. This is the measure of the average coordinates distance between the estimated source and the real one.

Root-mean-square-error (RMSE) on the distance is

(23) $$R_{RMSE}=\sqrt{\frac{1}{n}\sum_{i=1}^{n}\left[{\left({\hat{x}}_{i}-x\right)}^{2}+{\left({\hat{y}}_{i}-y\right)}^{2}\right]}$$

where *n* is the number of noisy measurements tested for each source location. 

All the geometries in Figure 8 are symmetrical. The reference sensor is located at (2, 2). CRLB provides the theoretical lower bound on the variance of any unbiased estimator [21]. It is also used in the comparisons. 

### 3.2. The Robustness

The drawback of iterative algorithm is that the result may not converge or fall into the local minima. The main factor is the poor quality of the noisy measurements. In the following sections, it will be found that in some cases the *Jεa*-based-FoPC and Taylor algorithms converge incorrectly, which affects the statistical results of the test. 

**Figure 9:**
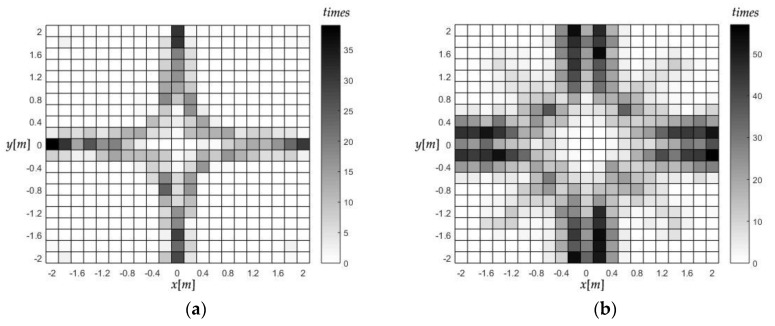
The divergence times distribution of Taylor and *Jεa*-based-FoPC. (**a**) *σ =* 0.03 m, (**b**) *σ =* 0.1 m.

Figure 9 is the divergence times distribution of the four algorithms. The divergence of Taylor and *Jεa*-based-FoPC were concurrent, while the *Jεe*-based-FoPC and this algorithm both converged well in all the tests. Notice that most of the divergence occurs near the crosshairs in the array, as Figure 9a. One of the TDOA measurements on these places is close to 0, which is more sensitive to errors and makes some matrix singular. So, it may be related to the dissatisfactory robustness of the two algorithms. When the measurement error becomes larger, as shown in Figure 9b, the divergence becomes more serious.

The input conditions were recorded when the wrong convergence happened and we take a part of these anomalies to observe in this section, as Table 1.

**Figure 10:**
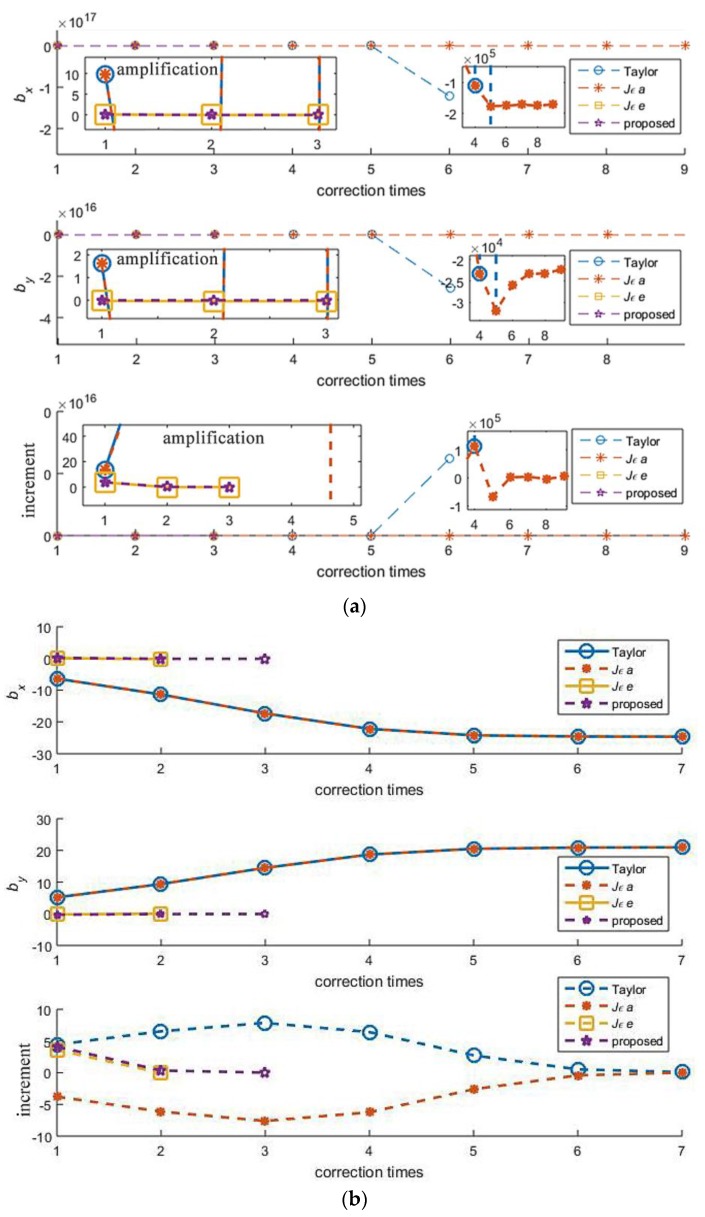
The biases to the true value and the increment to the initial value after each correction in the convergence process. (**a**) *R*_10_ = −0.043 m, *R*_20_ = −2.490 m, *R*_30_ = −2.433 m, ***p*** = (−2, 0)*^T^*, *σ* = 0.03 m, (**b**) *R*_10_ = 1.263 m, *R*_20_ = −0.226 m, *R*_30_ = −1.712 m, ***p*** = (−1, 1)*^T^*, *σ* = 0.1 m.

Under the input condition of the first line in Table 1, the iteration experience of these algorithms is shown in Figure 10a. The proposed approach and *Jεe*-based-FoPC have converged and stopped correcting after three times. While Taylor and *Jεa*-based-FoPC were of divergence from the correction beginning, the former was out of control and the latter was correcting in a wrong way.

Under the input condition of the sixth line in Table 1, the iteration experience of these algorithms is shown in Figure 10b. The proposed approach finished the correction after three times, *Jεe*-based-FoPC cost two times. Taylor and *Jεa*-based-FoPC seem to be converged, however, they failed into wrong results.

### 3.3. The Impact of the Source Position on Localization Accuracy

As above, the accuracy of TDOA localization is different when the localized source is in different places inside the array. If we exclude these singular results in statistics, the better features of Taylor and *Jεa*-based-FoPC arise, as shown in Table 2. 

**Figure 11:**
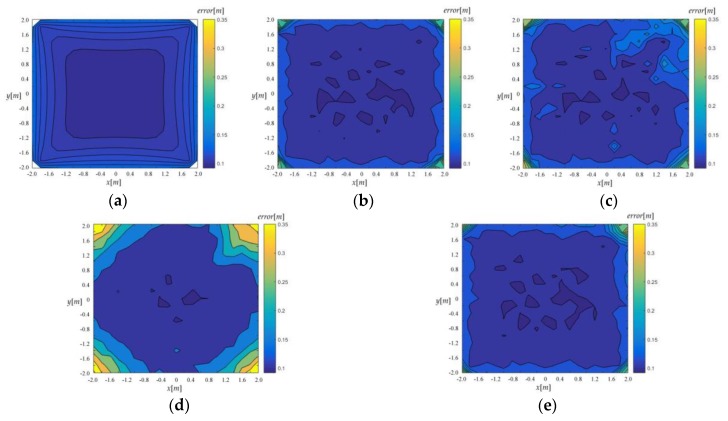
RMSE distribution, *σ* = 0.1 m. (**a**) CRLB, (**b**) Taylor, (**c**) *Jεa*-based-FoPC, (**d**) *Jεe*-based-FoPC, (**e**) the proposed algorithm.

In general, the localization accuracy is higher near the center of the array while it rapidly decreases near the BSs, which is illustrated in the CRLB distribution and the simulation results consistently. See in Figure 11.

**Figure 12:**
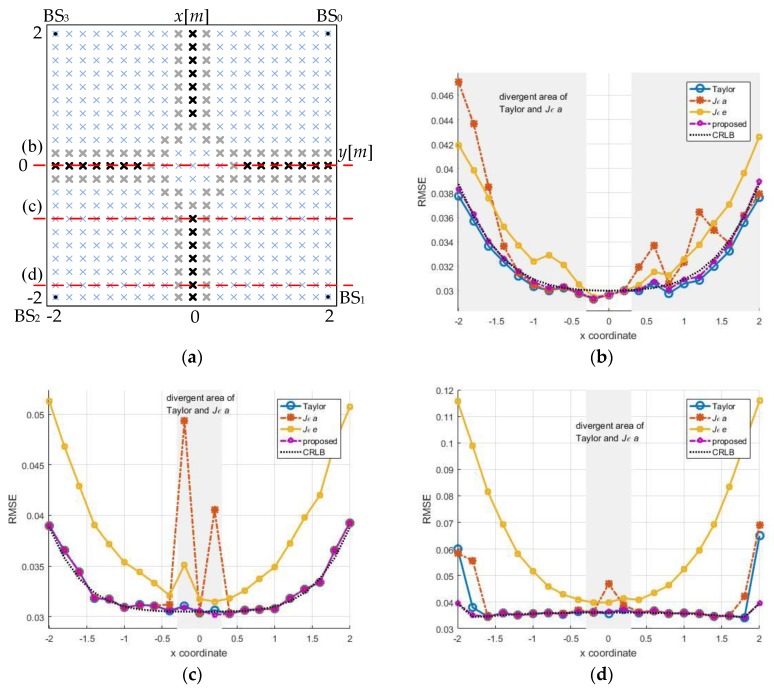
Profile analysis, *σ* = 0.03 m, the number of simulations is 2000. (**a**) Top view, (**b**) *y* = 0, (**c**) *y* = −0.8, (**d**) *y* = −1.8.

Three trajectory lines are drawn through the array along *y* = 0, *y* = −0.8, *y* = −1.8 and the source localization performance on these lines are analyzed using the four algorithms. At the corresponding positions in Figure 9, the phenomenon of multiple divergences was expectedly presented and those locations are marked with gray areas below. The statistical results excluding the singular value are shown in Figure 12.

If converge completely, Taylor approximates to CRLB. The *Jεa*-based-FoPC is similar to Taylor ideally. In Figure 12, some local minimum convergences have not been completely eliminated in statistics, which affected the results on some locations. The *Jεe*-based-FoPC and our method are robust and correctly converged inside the array. However, the *Jεe*-based-FoPC accuracy somewhat less than the others. In addition to robustness, the proposed algorithm is also close to CRLB as Taylor on accuracy.

### 3.4. The Impact of Measurement Noise on Localization Accuracy

Measurement noise are the other factors of the precision effect in TDOA localization. We conducted 2000 statistical tests at location (0, 0) with TDOA errors, ranging from 0.002 m to 0.4 m. The performance at location (0, 0) is best inside the array. The results are listed in Table 3. The RMSE of these algorithms are almost at the same and is proportional to *σ*. Notice that the statistical divergence times of Taylor and *Jεa*-based-FoPC also increase with TDOA errors, while the divergence never happens on *Jεe*-based-FoPC and the proposed algorithm at all. 

**Figure 13:**
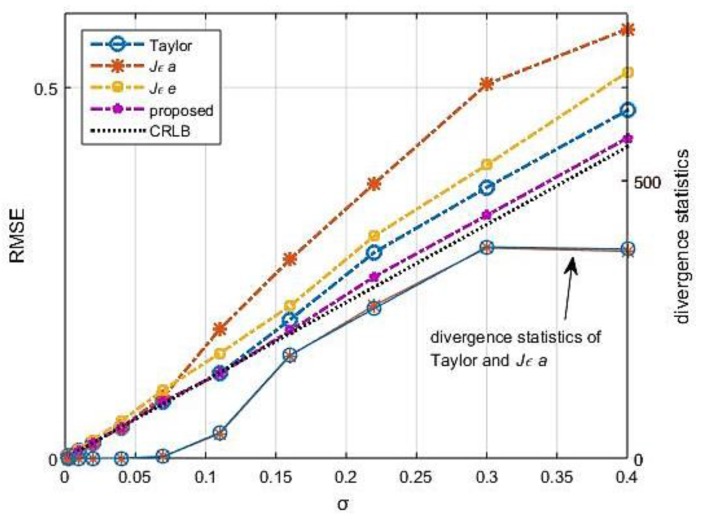
RMSE and the divergence times along the error changing, ***p*** = (1.2, 0.8)*^T^*.

Figure 13 shows the simulation results under the same conditions at location (1.2, 0.8). Although it can be concluded from Figure 9 that the convergence at location (1.2, 0.8) is better than others nearby, the divergence of Taylor and *Jεa*-based-FoPC is up to 380 times at most. Moreover, the differences in the anti-noise characteristics of the algorithms are evident in these cases. As we can see in Figure 13, the algorithm we proposed is always close to CRLB without divergence, it is robust distinctly.

## 4. Conclusions

In this paper, we have proposed a weighted least squares method solving the hyperbolic passive localization under the space-range frame. Unlike currently available cone-related ideas, this method uses cone tangent plane instead of cone surface locally and converts the nonlinear model into a linear model. In this way, the optimal solution can be obtained by WLS. In order to be robust, we add a vertical plane to constrain the estimations on the tangent without deviating from the cone. As same as some state-of-the-art algorithms, the correction process may be repeated until convergence to an optimal result. In theory, we resolve the weight value expressions of the two constrained planes, which effectively controls the convergence as we expect. The problem with the existing iterative algorithms is that they cannot achieve both precision and robustness at the same time. The cone tangent plane constraint algorithm provided a solution to this trouble in the paper.

We tested the performance of this algorithm and other analogs with a series of experiments. In the experiment, we found that classical Taylor algorithm and *Jεa*-based-FoPC algorithm often fail to converge under some conditions. High accuracy of these algorithms was observed through the data post-processing, which masked the real performance of the algorithms. These defects do not exist in this algorithm. Two cost functions proposed in FoPC have quite different properties. *Jεa*-based-FoPC is accurate but poor robustness, while *Jεe*-based-FoPC is the opposite. Tests in different locations illustrated the regional distribution of TDOA positioning accuracy; with the highest accuracy at the center while the poorer accuracy at the corners. Finally, in the noise increasing simulations, we observed the impact on positioning when the measurement error was great. Under these conditions, Taylor and *Jεa*-based-FoPC might converge abnormally even in the center. This impact is evident elsewhere and the differences in the performance of the algorithms are highlighted under loud noises. Notice that the proposed algorithm is always close to CRLB. In these experiments, the proposed algorithm has no risk of divergence and always guarantees the convergence and accuracy. It is also the significance of the newly designed algorithm with cone tangent plane constraint.

## Figures and Tables

**Table 1:** The condition records when the localization is the wrong convergent. Unit: m.

***σ***	**TDOA Measurements**			***p***	**Taylor ^1^**	***Jεa*^1^**	***Jεe*^1^**	**Proposed ^1^**
0.03	−0.043	−2.49	−2.433	(−2, 0)	divergent		−1.964	−1.964
							−0.038	−0.032
	−0.576	−0.876	−0.307	(−0.2, −0.4)	−59.604	−34639	−0.216	−0.211
					−102.87	−55.464	−0.408	−0.405
	2.498	2.559	0.048	(0, 2)	divergent		0.046	0.031
							2.037	2.037
	0.312	1.155	0.857	(0.6, 0.2)	−2.701	70.879	0.609	0.606
					−2.343	53.347	0.218	0.21
	0.012	2.46	2.449	(2, 0)	divergent		1.974	1.974
							0.009	0.007
***σ***	**TDOA Measurements**			***p***	**Taylor ^1^**	***Jεa*^1^**	***Jεe*^1^**	**Proposed ^1^**
0.1	1.263	−0.226	−1.712	(−1, 1)	−25.682	−25.682	−1.201	−1.111
					21.969	21.969	1.025	0.925
	−0.63	−0.26	0.382	(0.2, −0.4)	divergent		0.267	0.261
							−0.451	−0.449
	−1.745	−0.115	−1.296	(1, −1)	15.973	15.931	1.092	0.998
					−17.308	17.295	−1.183	−1.093
	−1.194	−2.127	−0.972	(−0.8, −0.8)	divergent		−0.704	−0.639
							−0.867	−0.812
	1.762	3.035	1.671	(1, 1.2)	13.457	7.969	1.224	1.007
					14.409	9.726	1.304	1.082

^1^ Note: listed below are biases ${\left(\begin{array}{cc} b_{x} & b_{y} \end{array}\right)}^{T}$, $b_{x}=x-\hat{x}$, $b_{y}=y-\hat{y}$.

**Table 2:** Average ***b*** and ***R***_rmse_ for the four algorithms. Unit: m.

Algorithm	*σ* = 0.03		*σ* = 0.1	
	*b*	*R_rmse_*	*b*	*R_rmse_*
Taylor	0.0025	0.0340	0.0103	0.1151
*Jεa*	0.0028	0.0355	0.0119	0.1191
*Jεe*	0.0031	0.0467	0.0120	0.1536
Proposed	0.0025	0.0359	0.0090	0.1189

**Table 3:** RMSE and the divergence times along the error changing, ***p*** = (0, 0)*^T^*.

*σ* (m)	0.002	0.01	0.02	0.04	0.07	0.11	0.16	0.22	0.3	0.4
RMSE (m)	0.002	0.010	0.020	0.040	0.070	0.109	0.160	0.221	0.299	0.392
Taylor	0	0	0	0	2	0	4	3	3	8
*Jεa*	0	0	0	0	2	1	4	3	4	9
*Jεe*	0	0	0	0	0	0	0	0	0	0
proposed	0	0	0	0	0	0	0	0	0	0
